# Cadmium Pathways in Snails Follow a Complementary Strategy between Metallothionein Detoxification and Auxiliary Inactivation by Phytochelatins

**DOI:** 10.3390/ijms21010007

**Published:** 2019-12-18

**Authors:** Martin Dvorak, Raimund Schnegg, Michael Niederwanger, Veronika Pedrini-Martha, Peter Ladurner, Herbert Lindner, Leopold Kremser, Reinhard Lackner, Reinhard Dallinger

**Affiliations:** 1 Institute of Zoology and Center for Molecular Biosciences Innsbruck, University of Innsbruck, Technikerstr. 25, A-6020 Innsbruck, Austria; 2 Institute of Clinical Biochemistry, Innsbruck Medical University, Biocenter, Innrain 80, A-6020 Innsbruck, Austria

**Keywords:** Mollusca, Gastropoda, slug, snail, cadmium tolerance, cadmium accumulation, cadmium binding selectivity, specificity, detoxification capacity, phytochelatin synthase

## Abstract

Metal detoxification is crucial for animals to cope with environmental exposure. In snails, a pivotal role in protection against cadmium (Cd) is attributed to metallothioneins (MTs). Some gastropod species express, in a lineage-specific manner, Cd-selective MTs devoted exclusively to the binding and detoxification of this single metal, whereas other species of snails possess non-selective MTs, but still show a high tolerance against Cd. An explanation for this may be that invertebrates and in particular snails may also synthetize phytochelatins (PCs), originally known to be produced by plants, to provide protection against metal or metalloid toxicity. Here we demonstrate that despite the fact that similar mechanisms for Cd inactivation exist in snail species through binding of the metal to MTs, the actual detoxification pathways for this metal may follow different traits in a species-specific manner. In particular, this depends on the detoxification capacity of MTs due to their Cd-selective or non-specific binding features. In the terrestrial slug *Arion vulgaris*, for example, Cd is solely detoxified by a Cd-selective MT isoform (AvMT1). In contrast, the freshwater snail *Biomphalaria glabrata* activates an additional pathway for metal inactivation by synthesizing phytochelatins, which compensate for the insufficient capacity of its non-selective MT system to detoxify Cd. We hypothesize that in other snails and invertebrate species, too, an alternative inactivation of the metal by PCs may occur, if their MT system is not Cd-selective enough, or its Cd loading capacity is exhausted.

## 1. Introduction

Snails (Gastropoda) represent one of the most species-rich phyla of the animal kingdom, playing an important role in marine, freshwater, and terrestrial ecosystems [1,2]. With a close dependence on their chemical environment and a mostly limited mobility, regulation and detoxification of non-essential toxic metals, like cadmium (Cd), is crucial for their growth and survival [3,4]. An important role in the detoxification of this metal is attributed to the wide-spread family of metallothioneins (MTs). These are mainly low-molecular sized proteins containing a high amount of Cys residues involved in binding of transition metal ions such as Zn^2+^, Cd^2+^, and Cu^+^ [5]. The amino acid sequence of MTs is characterized by repeat motifs of Cys residues such as Cys–X–Cys or Cys–X–X–Cys, where X is any amino acid except cysteine [6]. These motifs play a central role in formation of metal complexes [7], where metals are bound to the MT polypeptide in firm clusters of metal-thiolate bonds through the sulphur atoms provided by the various Cys residues [8]. Vertebrate and most other animal MTs exhibit a dumbbell-shaped three-dimensional structure with two metal binding domains, each containing a rigid core holding three to four metal ions [9]. Some snail species like *Littorina littorea* and *Pomatias elegans*, express three-domain MTs [10,11], which were not known until recently.

Owing to their unique metal binding features, MTs are primarily involved in capture and release of metal ions, serving physiological tasks related to storage, delivery, and inactivation of essential trace metals, as well as detoxification of non-essential metal ions [5,12,13,14]. In most animal MTs, the metal composition of metal thiolate clusters is promiscuous, although some difference in metal selectivity may exist between the protein domains [15,16,17]. Thus, under *in vivo* conditions most MTs exhibit a hetero-metallic and metamorphic composition, with low preferences for a defined metal species, if any at all [18,19,20]. Hence most metal-MT complexes may comprise a variable number of metal ions, even a mixture of different metal species (e.g., Zn^2+^ together with Cu^+^) [5]. In contrast, many species of Gastropoda possess metal-selective MT isoforms and variants, which form homometallic complexes with distinct transition metal ions. For example, some snails express Cu- and Cd-selective MT isoforms [21] that are prevalently devoted to physiological functions related to the respective metal ion species bound. Consequently, Cd-selective MT isoforms are involved in the inactivation and detoxification of Cd^2+^ ions in snail soft tissues [22]. The detoxification capacity of these isoforms is enhanced by the rapid Cd-induced transcription of their genes [10,23].

In addition to MT expression, some snails also possess the capacity to synthesize phytochelatins (PCs) in response to metal exposure [24]. PCs are cysteine-rich non-ribosomal peptides produced from glutathione (GSH) by the action of the enzyme phytochelatin synthase, which also plays a role in the catabolism of GSH conjugates in plants [25]. GSH is the major thiol compound produced by animals, plants, and bacteria. It is typically present at millimolar concentrations within cells and can constitute up to 90% of non-protein thiols [26]. Along with GSH, PCs are involved in protective intracellular functions such as oxidative stress defence, regulation of intracellular trace element concentrations and metal detoxification [27]. The general structure of PC peptides is (γ-GluCys)*_n_*Gly where *n* can assume a value between 2 to 11. They are wide-spread in plants [28], although more recent studies have documented their presence in different invertebrate animals, too [24,29], including the freshwater snail *Lymnaea stagnalis* [24]. The nematode *Caenorhabditis elegans* can detoxify Cd^2+^ and probably some other metal and metalloid ions by production of PCs [30], through the activity of phytochelatin synthase [31]. The *phytochelatin synthase* gene has a wide phylogenetic distribution across almost all of the animal tree of life. Available data until now suggest, however, that the occurrence of phytochelatin synthase is patchy, meaning that some members of particular taxonomic groups may contain the *phytochelatin synthase* gene, and some of those do synthesize PCs in response to potentially toxic elements [24].

How and if PCs in invertebrates interact with the function of MTs remains to be elucidated, and the temporal, spatial, and metal specificity of the two systems is still unknown. In invertebrate animals both systems coexist and both systems may function as scavengers in a first defence line of metal detoxification [32].

In our previous studies we have shown that Cd detoxification in snails is mainly based on the metal-binding capacity of MTs [33,34,35]. However, the efficiency of Cd detoxification may depend on the degree of their Cd selectivity. Some snails like marine littorinids and terrestrial helicids possess highly Cd-selective MTs with a high response and binding capacity against Cd exposure [21,23,36], whereas other species, particularly from freshwater environments, express poorly responsive, non-selective MTs that bind Cd^2+^ ions with a much lower degree of efficiency [37]. In some of these species like *Lymnaea stagnalis*, apparently both detoxification systems (MTs and PCs), co-exist [24,38]. The potential role of PCs and their interaction with the MT system, however, has so far not been considered. In the present study we test the hypothesis that both detoxification systems (MTs and PCs) complement each other in detoxification of Cd, depending on the degree of Cd response and selectivity of the respective MT isoforms. We compare two species of gastropods, *Arion vulgaris* and *Biomphalaria glabrata*, and their response to Cd exposure. *Arion vulgaris* possesses an inducible Cd-selective MT (AvMT1) [39], while the second species relies on a non-selective MT that is hardly inducible by Cd exposure [37,40]. We provide evidence that in snails, PCs are ready to take over protection against Cd impact, if the detoxification capacity of MTs becomes deficient due to lacking metal binding selectivity or oversaturation of their binding capacity.

## 2. Results and Discussion

### 2.1. Species-Specific Differences in Cd Uptake and the Role of Metallothioneins

**Figure 1:**
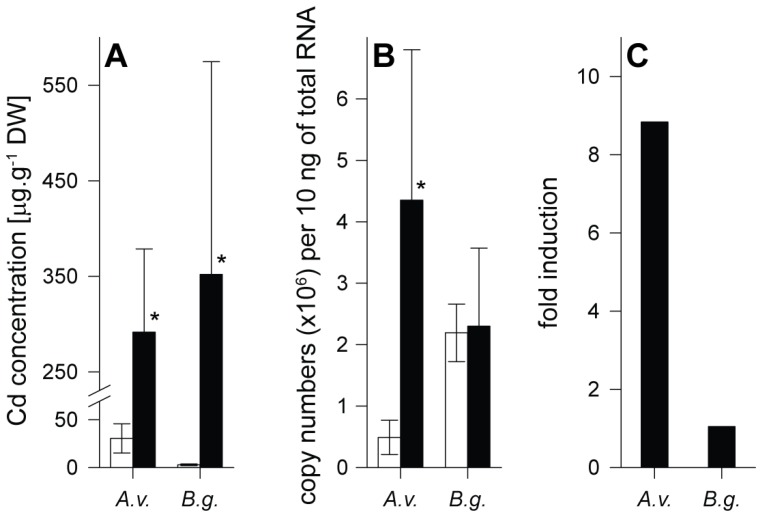
Comparison of control (white columns) and Cadmium (Cd) exposed (black columns) individuals of *Arion vulgaris* (*A.v.*) and *Biomphalaria glabrata* (*B.g.*). (**A**) Cd concentrations expressed in µg per g dry weight (DW), shown in midgut gland of *A.v*. and *B.g*. exposed to Cd over a period of 15 days (*A.v*.) and 14 days (*B.g*.) (means and standard deviations, *n* = 3–4). (**B**) Transcriptional upregulation of *MT* genes of *Arion vulgaris* (*AvMT1*, GenBank: MF155618.1) and *Biomphalaria glabrata* (GenBank: XM_013225031), expressed as mRNA copy numbers per 10 ng of total RNA after Cd exposure over a period of 15 days (*A.v.*) and 14 days (*B.g.*) (means and standard deviations, *n* = 5). (**C**) Transcriptional upregulation of *MT* genes of *A.v.* and *B.g.*, expressed as -fold induction (mean values, derived from **B**). The asterisks indicate significant differences compared to respective control values (Holm–Sidak method of all pairwise multiple comparisons) (* *p* ≤ 0.05).

In spite of the different experimental regimes and uptake routes through food or water in the terrestrial slug *Arion vulgaris* and the freshwater snail *Biomphalaria glabrata*, both species strongly accumulated Cd in their midgut gland upon metal exposure (Figure 1A), reaching concentrations of about 290 (*Arion vulgaris*) and 350 µg Cd/g dry wt. (*Biomphalaria glabrata*), respectively. Compared to Cd control values of about 27 µg Cd/g dry wt. in *Arion vulgaris* and 3 µg Cd/g in *Biomphalaria glabrata*, this corresponds to metal accumulation factors in the midgut gland of about 11 and 116, respectively. At the same time, no mortality was observed in the two species, confirming their high tolerance and detoxification capacity against this highly toxic metal [37,39].

In spite of the much stronger Cd accumulation in *Biomphalaria glabrata*, only in *Arion vulgaris* a significant transcriptional upregulation (9-fold) of the respective Cd-selective *AvMT1* gene occurs (GenBank: MF155618, [39]). In contrast, no detectable upregulation was observed for the unspecific *MT* gene of *Biomphalaria glabrata* (GenBank: KT697617, [37]) (Figure 1B,C).

**Figure 2:**
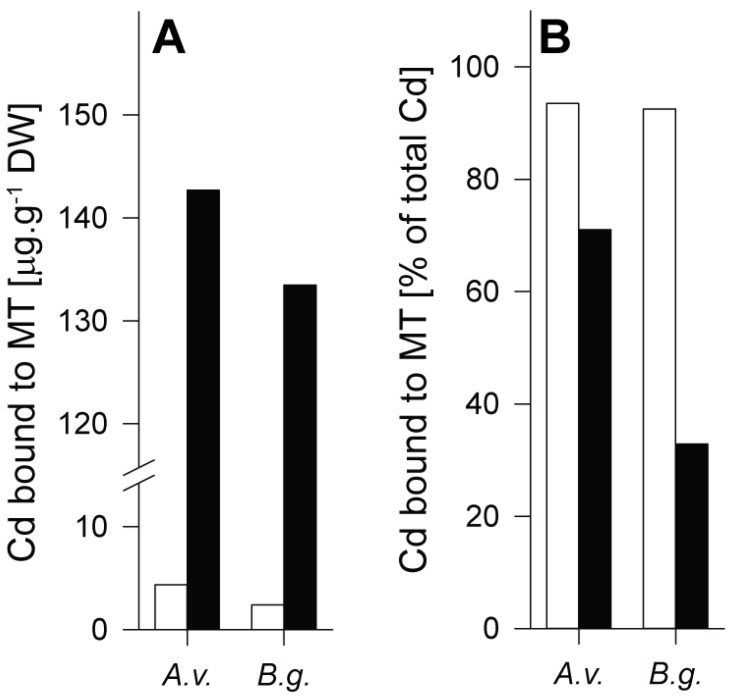
Cadmium (Cd) concentrations related to metallothionein (MT) fractions after gel chromatography separation from midgut gland extracts of control (white columns) and Cd-exposed (black columns) *Arion vulgaris* (*A.v*.) and *Biomphalaria glabrata* (*B.g*.), expressed as Cd bound to MT (in µg/g DW, as above) (**A**); and as percentage fraction (%) of total Cd bound to MT (**B**).

In both species, the Cd amount associated to MT under control conditions was below 5 µg/g dry wt. After Cd exposure, however, about 135–145 µg of the metal in the midgut gland of the two species were bound to MT (Figure 2A). If referred to the total Cd concentration in this organ, the percentage fraction of Cd bound to MT after metal exposure largely differed between the two species. In *Arion vulgaris*, a predominant portion of the metal (75%) was bound to the metal-specific MT (AvMT1), whereas only 32% of the metal was associated with the non-specific MT in the midgut gland of *Biomphalaria glabrata* (Figure 2B). Thus concomitantly with the differential Cd-dependent *MT* gene upregulation pattern of the two species (Figure 1B,C), the Cd-selective AvMT1 of *Arion vulgaris* appears to possess a much higher detoxification potential for Cd than the unspecific MT of *Biomphalaria glabrata*. Since both species exhibit a considerable tolerance towards Cd upon metal exposure (see above), this suggests that the lacking inactivation capacity for Cd by the MT system in *Biomphalaria glabrata* must be compensated by an additional mechanism of detoxification.

### 2.2. Transcriptomic Analyses of Gastropod Phytochelatin Synthase mRNA Transcripts

Following the discovery of PCs in a snail [24], a primary candidate for such an additional mechanism would be scavenging of Cd by PCs. While MTs are metal-binding proteins that are encoded by their respective genes, PCs are peptides that are metabolically synthesized through the activity of the enzyme phytochelatin synthase. A screening of our transcriptomic databases revealed the presence of *phytochelatin synthase* genes in all species for which transcriptomes were available. In addition, a search in NCBI databases uncovered additional snail *phytochelatin synthase* genes (Table 1). Overall, this suggests that *phytochelatin synthase* genes are present in probably all major gastropod clades. However, Table 1 does not specify whether the respective phytochelatin synthase enzymes are actually active.

**Figure 3:**
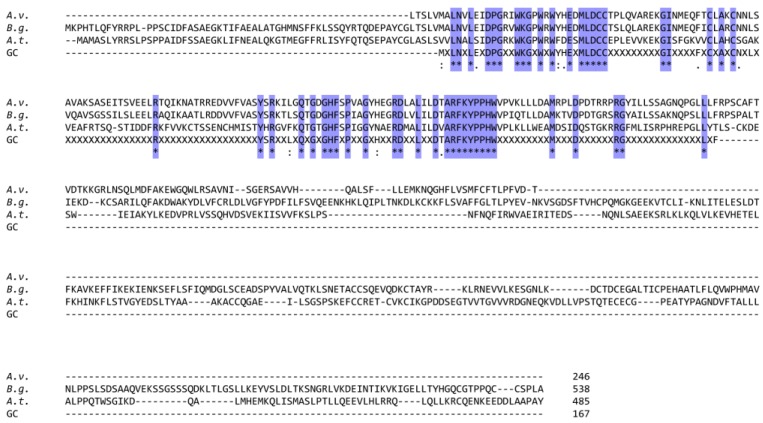
Comparison of amino acid sequences of phytochelatin synthase of *Arion vulgaris* (*A.v.*) (incomplete), *Biomphalaria glabrata* (*B.g.*), and *Arabidopsis thaliana* (*A.t.*), in addition to the respective all available gastropod consensus sequence (GC). Conserved amino acid positions are shadowed in blue. The symbol X means any non-conserved amino acid residue. The symbol “-” stands for alignment gaps.

Phytochelatin synthase was first characterized in plants [42]. When comparing the translated amino acid sequences of phytochelatin synthase from the plant *Arabidopsis thaliana* (GenBank: AAD41794.1, [43]) with those of the slug *Arion vulgaris* and the snail *Biomphalaria glabrata*, a certain degree of homology is obvious. This becomes also apparent upon highlighting the resulting consensus sequence between *Arabidopsis thaliana* and all available gastropods (Figure 3). On the other hand, the variability within the deduced gastropod phytochelatin synthase sequences follows largely the diversity in gastropod systematics (Figure S1).

### 2.3. Metallothioneins versus Phytochelatins

Both MTs and PCs provide sulphur atoms for metal ion binding through their Cys residues. In Table 2, Cys concentrations in MTs or PCs, expressed as molar Cys equivalents theoretically available for Cd^2+^ binding, are reported. The data were calculated assuming a binding ratio of Cys: Cd^2+^ of 3:1 in the Cd-selective AvMT1 of *Arion vulgaris* [39], 1.83:1 in the unspecific MT of *Biomphalaria glabrata* [40], and 2:1 in PCs [44,45]. If we consider that the unspecific MT of *Biomphalaria glabrata* forms, owing to its non-specific binding features for Cd^2+^, mixed metal complexes involving other metal ions like the divalent Zn^2+^ and the monovalent Cu^+^, too [40], it must be assumed that the real availability of Cys for Cd^2+^ binding in this MT may even be lower than that reported in Table 2. Moreover, the low fraction of total Cd^2+^ associated with MT in the midgut gland of *Biomphalaria glabrata* (see Figure 2B) shows that a considerable amount of the metal pool in this snail is kept apart from MT, and would need, if any, additional mechanisms for its detoxification. To compensate for the missing detoxification capacity by MT, *Biomphalaria glabrata* seems to rely on PC synthesis. Hence, in contrast to *Arion vulgaris*, a considerable fraction of Cd^2+^ in *Biomphalaria glabrata* may be buffered by Cys equivalents provided by PCs (Table 2).

**Figure 4:**
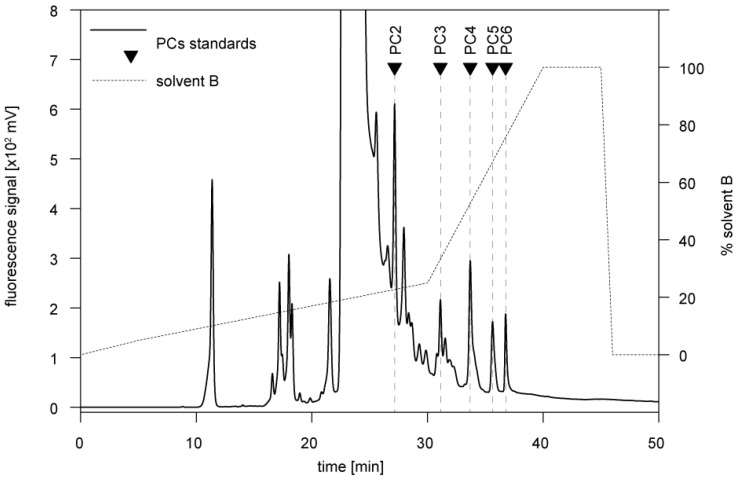
Reversed phase HPLC of a phytochelatin (PC) standard mixture (PC2–PC6) derivatized with monobromobimane, and confirmed by mass spectrometry. Elution times of individual PCs are represented by triangles.

PCs are polymerized in the cellular metabolism from GSH to peptides with different chain lengths (mainly PC2–PC6) through the activity of phytochelatin synthase. In our study, the presence of PCs in the two snail species was assessed through HPLC elution after derivatization to fluorescent thiol-bimane derivatives. As shown by the respective HPLC profile of a PC standard mixture (containing PC2, PC3, PC4, PC5, and PC6) this method allowed detecting all single species between PC2 to PC6 as separate peaks (Figure 4).

**Figure 5:**
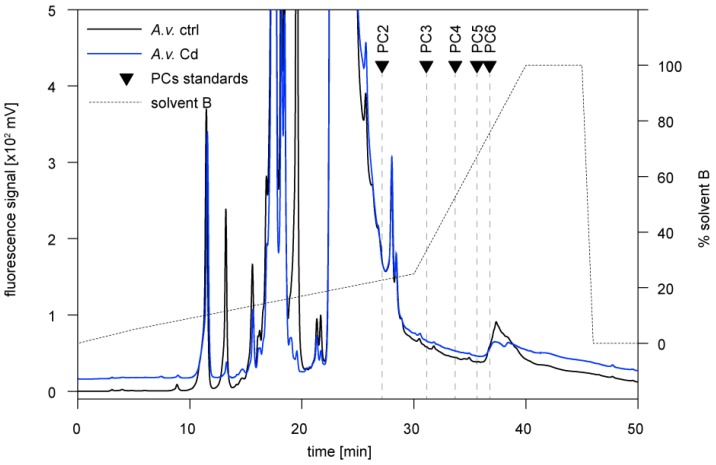
Reversed phase HPLC of *Arion vulgaris* (*A.v.*) midgut gland homogenates derivatized with monobromobimane for detection of phytochelatins. Black line: non-exposed animal (ctrl); blue line: Cd-exposed animal for 15 days (Cd). Triangles indicate elution times of standard phytochelatins (PC2–PC6).

**Figure 6:**
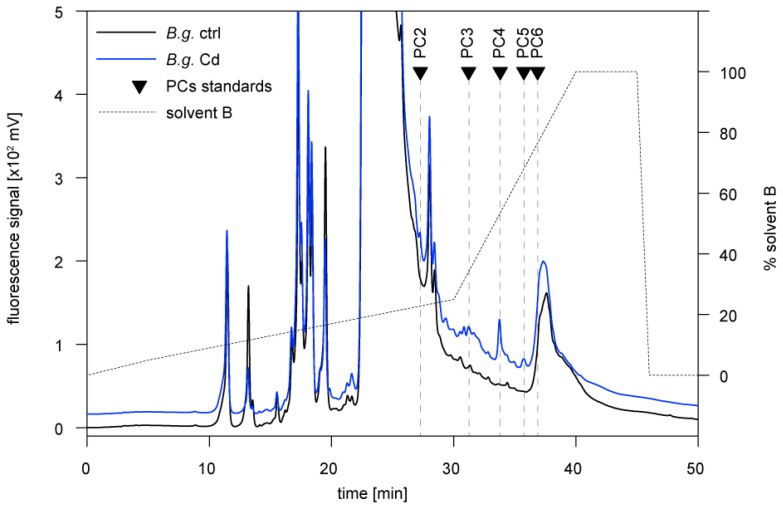
Reversed phase HPLC of *Biomphalaria glabrata* (*B.g.*) homogenates derivatized with monobromobimane for detection of phytochelatins. Black line: non-exposed animal (ctrl); blue line: Cd-exposed animal for 14 days (Cd). Triangles indicate elution times of standard phytochelatins (PC2–PC6).

Although *Arion vulgaris* accumulates Cd (Figure 1A), only traces of PC2, if any, were observed in the midgut gland of control and Cd-exposed slugs (Figure 5). Instead, all Cys equivalents available for Cd^2+^ binding and detoxification were provided by the Cd-selective AvMT1 protein [39] (Table 2). The situation was different in *Biomphalaria glabrata*. Apart from PC2, no other PCs were found in homogenates of control *Biomphalaria glabrata*. Upon exposure to Cd, however, *Biomphalaria glabrata* did not induce expression of MT in its midgut gland (Figure 1B,C), and only about 35% of Cd^2+^ were associated with the non-specific MT (Figure 2B). Instead, the snail produced considerable amounts of PCs (Figure 6), reaching altogether molar concentrations of up to 0.55 µmol/g dry wt. Cys equivalents potentially available for Cd^2+^ binding (Table 2).

As shown in Table 1, the *phytochelatin synthase* gene was identified in both, *Arion vulgaris* and *Biomphalaria glabrata*, as well as in most other gastropod genomes or transcriptomes studied so far. The *phytochelatin synthase* gene and its respective transcript seem to be quite conservative and widely distributed throughout the Gastropods (see Figure 3 and Figure S1). However, this does not imply that the phytochelatin synthase enzyme is present, functional or actually involved in Cd detoxification. In a recent article by Bundy and colleagues [29], the authors suggested that there is a functional relationship between MTs and PCs in a response-time depending manner.

Our data provide a more extended hypothesis and indicate that in midgut gland cells of snails, GSH is only polymerized to PC peptides in the presence of available Cd^2+^ and other metal ions in a free form, irrespective of whether this happens due to a delayed response pattern or by exhaustion of the MT detoxification capacity. Indeed, such an assumption would explain the differences found in our study between lacking PC synthesis in *Arion vulgaris*, in contrast to *Biomphalaria glabrata*. In this species and probably in many other snails with non-specific MTs [37,40], the detoxification capacity of the MT system may become overstrained, because Cd^2+^ ions are bound to the protein with poor selectivity. Hence, upon exposure to Cd beyond a certain threshold, alternative Cd^2+^ inactivation by complexation to PCs is highly sensible. In the hierarchy of the two detoxification mechanisms, the MT system will probably take the lead over PC synthesis. Different gastropod species express their respective MT isoforms with different expression patterns and intensity depending, among others, on the metal-binding selectivity of their MT isoforms. Pulmonate land snails, for example, possess distinct MT isoforms with metal binding selectivities for different metals [21,46]. Two MT isoforms (AvMT1 and AvMT2) are also present in *Arion vulgaris*. The AvMT1 isoform is inducible by Cd, being mainly involved in selective detoxification of this metal, whereas the isoform AvMT2 seems to play a minor role in Cu binding, but is expressed in the slug’s midgut gland only in trace amounts, if any [39]. In contrast to terrestrial helicids, many freshwater snails (including *Biomphalaria glabrata*) do not possess metal-inducible *MT* genes, nor are the expressed proteins Cd-selective. Apart from Cd selectivity, all MTs (including those of gastropods), bind the metal with a very high affinity, featuring stability constants for Cd^2+^ of about 2 × 10^16^ (at pH7) [18]. In contrast to MTs, the stability constant of PC-Cd^2+^ complexes is much lower [47]. Hence, the Cd-scavenging function of PCs becomes masked in species where all available Cd is inactivated by MTs with a high selectivity for this metal. According to this assumption, inactivation of Cd by PCs would occur only in species possessing MTs with a low induction potential and/or without Cd-binding selectivity. Thus overall, phytochelatin synthase becomes involved in a line of detoxification processes where Cd-selective MTs are insufficiently available, lacking, or overstrained in their binding capacity.

### 2.4. Methodological Considerations

Several methods have been reported in the literature to measure concentrations of MTs or metallothionein-like proteins (MTLPs) in animal tissues. One of the most commonly used methods relies on the assessment of thiol groups provided by Cys residues present in MTs or MTLPs by specific chemical reagents. For example, 5,5-dithiobis-2-nitrobenzoic acid (DTNB, Ellmans reagent) gives a yellow colour with SH-containing compounds. In many approaches, the respective tissue is homogenized and fractionated via heat denaturation, organic solvent extraction, and ultrafiltration. By any means this yields low molecular weight protein fractions where the SH- contents are measured photometrically. This value is synonymized with the MT or MTLP concentration of the tissue [48,49,50]. However, also PCs give a positive reaction with DTNB. Thus, PCs may hide behind the MTLP measurement with DTNB. Therefore, specificity of this method strongly depends on the efficiency of the organic solvent fractionation. Hence the actual measurement relies on the assumption that all MTs are found in the protein pellet after organic solvent treatment.

Differential pulse polarography (DPP) may be seen as an alternate method to measure SH- groups in a solute. Depending on the pre-treatment of the sample and the degree of purification this method may give a more or less true measure for the MT concentration in the sample. It has been used to measure the animals’ response to environmental pollution [51].

Silver saturation may be used to measure metal binding capacity of homogenates and of MTs [52]. Shortly, all compounds binding metals are saturated with silver, thereby replacing all other metals from the active sites. After removing excess silver and fractionation of the homogenate, silver is usually measured by atomic absorption spectroscopy (AAS). Depending on the quality of this separation, the results may be assigned to MTs or MTLPs. This method probably does not distinguish between MTs and PCs. However, it may be useful to detect SH-linked detoxification processes induced by environmental stressors. In the freshwater snail *Lymnaea stagnalis*, an induction of so-called MTLPs was observed only at very high waterborne concentrations of Cd (1000 µg L^−1^ [53]), and a similar pattern of induction was also reported from the dogwhelk *Nucella lapillus* [54,55].

A special case of a metal saturation method is the quantification of MT concentrations by the so-called Chelex assay [56], in which pre-purified MT extracts are saturated with Cd and quantified through the known stoichiometric Cd:MT ratio of snail MTs (6:1) after removal of excess amounts of Cd by Chelex-100 resin [34]. This method does only work for Cd-selective MTs (as in terrestrial snails), and has been successfully applied for environmental monitoring of Cd pollution in soil habitats [57].

Copper replacement of other metals in MTs is a method similar to the above mentioned silver saturation. The CuMT generated by this method exhibits a specific fluorescence, which may be used to quantify MTs in tissues [58]. Alternatively, a Cu saturation method called TTM assay may be applied for quantification of Cu-selective MTs, with calculation of CuMT through the known Cu:MT ratio of Cu-specific MT in snails [34].

An antibody against MT-Class I (Cat. #18-0133, Zymed, South San Francisco, CA, USA) was used to quantify MTs in the mud snail *Ilyanassa obsoleta* (now *Tritia obsoleta*) by ELISA. Upon exposure to Cd at low (0.5 µM) or high (50 µM) concentrations in brackish water, a huge increase of MT was observed in the whole soft body [59].

As MTs are proteins with a distinct molecular mass, all quantification methods that rely on protein purification prior to quantification may provide more reliable data. For example a simple gel chromatography fractionation followed by measurement of metals in the appropriate fractions gives a suitable approximation of MT concentrations [34,39]. In this case, measurement of the SH- concentration by any of the above mentioned methods should also come to acceptable results [51].

Finally, molecular methods also offer several possibilities to study the expression and induction of the respective *MT* genes by environmental factors [37,39,60].

## 3. Material and Methods

### 3.1. Transcriptome Generation and Screening for Phytochelatins Synthase Genes

For generating transcriptomes from different gastropods, isolated total RNA from midgut gland [39,40] was sent to the Duke Center for Genomic and Computational Biology (GBC, Duke University, Durham, NC, USA). The RNA was subjected to 125 bp paired-end Illumina sequencing. Raw data were assembled at the Institute of Zoology (University of Innsbruck) using Trinity Version v2.1.1 (GitHub Inc., San Francisco, CA, USA) [61] and provided for analysis on a local TBlast page. For details see Dvorak et al. (2018), Schmielau et al. (2019) [11,39].

All available transcriptomes of gastropods were searched for putative phytochelatin synthase transcripts. We confirmed the presence of the conserved phytochelatin synthase domain of the uncharacterised phytochelatin synthase mRNA sequence (GenBank: XM_013214798) in *Biomphalaria glabrata* by degenerated primers (forward: TGGAAAGGAYCATGGMGATG; reverse: CCARTGAGGTGGRTATTTAA) (Figure S1) and used it as a template for BLAST analysis in the NCBI database [62,63].

### 3.2. Cd Exposure and Tissue Analysis

*Arion vulgaris* slugs were collected in close vicinity of Innsbruck and kept on moistened garden soil (18 °C, 80% humidity, and 12 hr light/dark cycles). They were fed on Cd-enriched lettuce leaves for 15 days [39]. Aliquots of midgut gland were taken for MT mRNA quantification by quantitative real-time polymerase chain reaction (qRT PCR). Other aliquots were used for MT separation by gel chromatography and for determination of Cd tissue accumulation by atomic absorption spectroscopy (AAS). For details see Dvorak et al. (2018) [39].

*Biomphalaria glabrata* snails out of a laboratory culture at the Institute of Zoology, University of Innsbruck, were kept in freshwater aquarium tanks (25 °C, 12 h light/dark cycles) and exposed to a nominal Cd concentration of 75 µg·L^−1^ [37]. *MT* gene upregulation and Cd accumulation were measured in the midgut gland by respective methods, for details see Niederwanger et al. (2017) [37].

### 3.3. Determination of Phytochelatins

Determination of phytochelatins was based on a modified protocol of HPLC with detection of fluorescent thiol-bimane derivatives as described by Kawakami et al. (2006) [27]. Briefly, homogenized midgut gland in 0.1 M TFA, 10 mM EDTA, and 10 mM TCEP (1:3 *w*/*v*) was centrifuged at 13,200 rpm for 20 min at 4 °C. Fifty microliters of flow-through was pre-derivatized with 150 µL of 0.1 M NH_4_HCO_3_ and 50 µL 0.1 M TCEP in 0.1 M NH_4_HCO_3_ for 1 h at room temperature. The disulphide bonds were converted to sulfhydryls by adding 615 µL HEPES buffer and 25 µL 20 mM TCEP and incubation for 10 min at 45 °C, followed by addition of 10 µL of 250 mM monobromobimane and derivatization for 45 min at 45 °C in the dark. Derivatization was terminated by adding 100 µL 1M methanesulfonic acid. The derivatized sample was centrifuged for 1 min at 13,200 rpm, and 100 µL of supernatant was applied to reverse phased HPLC with a LiChrospher 100 RP-18 (5 µm) column (Merck, Kenilworth, NJ, USA) thermostatized at 40 °C. The thiol-bimane derivates were eluted on a linear gradient of buffer A consisting of 0.1% TFA and buffer B consisting of 0.1% TFA in acetonitrile (0%–5% B for 0–5 min, 5%–25% B for 5–30 min, and 25%–100% for 30–40 min) at a flow rate 1 mL·min^−1^. Fluorimetric detection was achieved with 380 nm (excitation) and 470 nm (emission) wavelengths. The calibration curve was obtained with a mixture containing 5 µg of each (PC2–PC6) of phytochelatin standards derivatized in the same way as the samples. HPLC fractions were manually collected and provided for MS analysis to confirm individual phytochelatin species.

### 3.4. Mass Spectroscopy

Samples were analysed using an UltiMate 3000 RSCLnano-HPLC system coupled to a Q Exactive HF mass spectrometer (both Thermo Scientific, Bremen, Germany) equipped with a Nanospray Flex ionization source. The PCs were separated on a homemade fritless fused-silica micro-capillary column (100 µm i.d. × 280 µm o.d. × 19 cm length) packed with 2.4 µm reversed-phase C18 material. Solvents for HPLC were 0.1% formic acid (solvent A) and 0.1% formic acid in 85% acetonitrile (solvent B). The gradient profile was as follows: 0–4 min, 4% B; 4–57 min, 4–35% B; 57–62 min, 35–100% B, and 62–67 min, 100% B. The flow rate was 300 nL·min^−1^. The Q Exacitve HF mass spectrometer was operating in the data dependent mode selecting the top 10 most abundant isotope patterns from the survey scan with an isolation window of 1.6 mass-to-charge ratio (*m*/*z*). Survey full scan MS spectra were acquired from 200 to 2000 *m*/*z* at a resolution of 60,000 with a maximum injection time (IT) of 120 ms, and automatic gain control (AGC) target 1e6. The PCs were identified by comparing the retention time and mass of the detected peaks in the chromatograms of standard PCs and samples.

### 3.5. Calculation of Cd Fractions Bound to MT and to PC

Cadmium associated to MTs was calculated as a sum of Cd concentrations from GPC fractions in the MT range per g of dry weight tissue. For Cys equivalents available for Cd binding we assumed 100% saturation in *Arion vulgaris* CdMT, resulting in Cys:Cd ratio of 3:1. In unspecific *Biomphalaria glabrata* MT we assumed up to 66% saturation resulting in 1.83:1 Cys:Cd ratio. Similarly for PCs, values refer to sum of individual PC concentrations per g of dry weight tissue.

### 3.6. Statistics

Relevant statistical analysis was performed in Sigmaplot 12.5 (SYSTAT software, San Jose, CA, USA)(for details see Dvorak et al. (2018) [39] and Niederwanger et al. (2017) [37]).

## 4. Conclusions

In most animals, MTs play a pivotal role in regulation of transition metals and detoxification of Cd, in particular. In spite of this, the actual detoxification mechanisms for Cd in different animal species may follow distinct pathways, sometimes through additional inactivation of this metal by phytochelatins. In the present study, we show exemplarily that in gastropods, the pathway of Cd inactivation depends on the detoxification capacity of MTs, in combination with an auxiliary metal complexation by phytochelatins. Upon Cd exposure, the terrestrial slug *Arion vulgaris* relies exclusively on Cd detoxification by means of a Cd-selective MT isoform (AvMT1) expressed in its midgut gland. In contrast, Cd-exposed individuals of the freshwater snail *Biomphalaria glabrata*, cope with the metal stress by synthesizing, in addition to an unspecific MT, phytochelatins. We conclude that the activation of the phytochelatin system for Cd inactivation in snails apparently depends on the Cd binding efficiency and metal selectivity of their MTs. We hypothesize that this pattern could be of general importance for many other invertebrate lineages, too.

## Figures and Tables

**Table 1:** The presence of putative phytochelatin synthase mRNAs in transcriptomes of different gastropod species. Complete sequences include start and stop codon.

Id	Name	Clade	Habitat	Source	Reading Frame
M.c.	*Marisa cornuarietis*	Caenogastropoda	Freshwater	This study	Complete
P.b.	*Pomacea bridgesii*	Caenogastropoda	Freshwater	This study	Complete
P.c.	*Pomacea canaliculata*	Caenogastropoda	Freshwater	NCBI XP_025099563.1	Complete
A.h.	*Anentome* *helena*	Caenogastropoda	Freshwater	This study	Complete
P.e.	*Pomatias elegans*	Caenogastropoda	Terrestrial	This study	Missing stop codon
A.c.	*Aplysia californica*	Heterobranchia	Marine	NCBI XM_005110788.2	Complete
E.c.	*Elysia crispata*	Heterobranchia	Marine	This study	Complete
B.g.	*Biomphalaria glabrata*	Heterobranchia Hygrophila	Freshwater	NCBI XM_013214798.1	Complete
G.t.	*Galba truncatula*	Heterobranchia Hygrophila	Freshwater	Romiguier et al. (2014) [41]	Complete
L.m.	*Limax maximus*	Heterobranchia Stylommatophora	Terrestrial	This study	Complete
C.a.	*Cornu aspersum*	Heterobranchia Stylommatophora	Terrestrial	This study	Complete
H.p.	*Helix pomatia*	Heterobranchia Stylommatophora	Terrestrial	This study	Complete
A.b.	*Alinda biplicata*	Heterobranchia Stylommatophora	Terrestrial	This study	Complete
A.v.	*Arion vulgaris*	Heterobranchia Stylommatophora	Terrestrial	NCBI PRJEB7891	Missing start and stop codon
P.v.	*Patella vulgata*	Patellogastropoda	Marine	This study	Complete
N.p.	*Neritina pulligera*	Neritimorpha	Freshwater	This study	Complete
T.l.	*Titiscania limacina*	Neritimorpha Cycloneritimorpha	Marine	NCBI PRJNA253054	Missing stop codon

**Table 2:** Equivalents of cysteine (Cys) expressed in µmol per g of dry weight tissue, available for Cd binding in metallothioneins (MT) and phytochelatins (PC).

Species	Treatment	Equivalents of Cys [µmol/g Dry wt.] Available for Cd^2+^ Binding	
		MT	PC
*Arion vulgaris*	Control	0.12	<0.01
	Cd-exposed	3.81	<0.01
*Biomphalaria glabrata* ^1^	Control	0.04	0.12
	Cd-exposed	2.18	0.55

^1^ In *Biomphalaria glabrata* MT 1 Cd = 1.83 Cys which equals 66% of Cys available for Cd binding.

## Supplementary Materials

Supplementary materials can be found at https://www.mdpi.com/1422-0067/21/1/7/s1. Figure S1: Alignment of diverse gastropods phytochelatin synthase protein sequences. Conserved regions are highlighted in red (throughout all clades), orange (Caenogastropoda) and pink (Heterobranchia). Following species are listed: *Marisa cornuarietis* (*M.c.*), *Pomacea bridgesii* (*P.b.*), *Pomacea canaliculata* (*P.c.*), *Anentome helena* (*A.h.*), *Pomatias elegans* (*P.e.*), *Aplysia californica* (*A.c.*), *Elysia crispata* (*E.c.*), *Biomphalaria glabrata* (*B.g.*), *Galba truncatula* (*G.t.*), *Limax maximus* (*L.m.*), *Cornu aspersum* (*C.a.*), *Helix pomatia* (*H.p.*), *Alinda biplicata* (*A.b.*), *Arion vulgaris* (*A.v.*), *Patella vulgata* (*P.v.*), *Neritina pulligera* (*N.p.*), *Titiscania limacina* (*T.l.*). Verified presence of *Biomphalaria glabrata* conserved phytochelatin synthase domain by PCR shown in black rectangle.
