# Supplementary material for: Cadmium Pathways in Snails Follow a Complementary Strategy between Metallothionein Detoxification and Auxiliary Inactivation by Phytochelatins
Source: Int J Mol Sci. 2019 Dec 18;21(1):7. doi: 10.3390/ijms21010007 (PMC6981842; doi:10.3390/ijms21010007)
Supplement: Supplementary file 1 [file ijms-21-00007-s001.pdf]

Supplementary materials

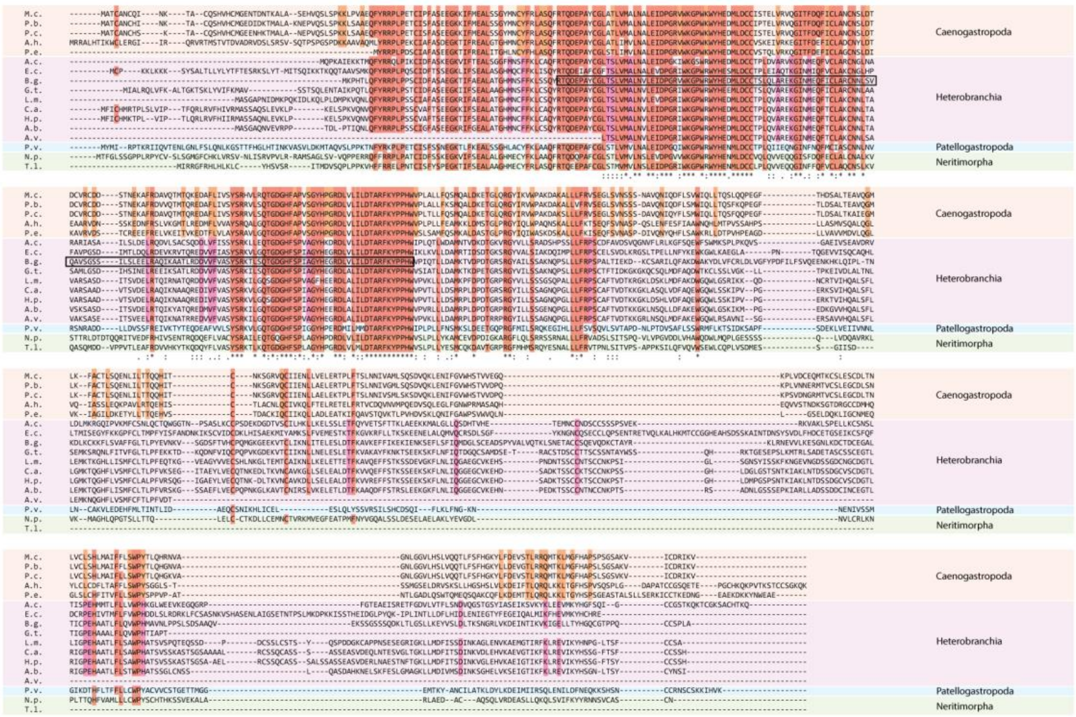

Figure S1: Alignment of diverse gastropods phytochelatin synthase protein sequences. Conserved regions are highlighted in red (throughout all clades), orange (Caenogastropoda) and pink (Heterobranchia). Following species are listed: *Marisa cornuarietis* (*M.c.*), *Pomacea bridgesii* (*P.b.*), *Pomacea canaliculata* (*P.c.*), *Anentome helena* (*A.h.*), *Pomatias elegans* (*P.e.*), *Aplysia californica* (*A.c.*), *Elysia crispata* (*E.c.*), *Biomphalaria glabrata* (*B.g.*), *Galba truncatula* (*G.t.*), *Limax maximus* (*L.m.*), *Cornu aspersum* (*C.a.*), *Helix pomatia* (*H.p.*), *Alinda biplicata* (*A.b.*), *Arion vulgaris* (*A.v.*), *Patella vulgata* (*P.v.*), *Neritina pulligera* (*N.p.*), *Titiscania limacina* (*T.l.*). Verified presence of *Biomphalaria glabrata* conserved phytochelatin synthase domain by PCR shown in black rectangle.
